# Femoral–facial syndrome in a Black Bantu African preterm infant: a case report

**DOI:** 10.1186/s13256-025-05181-7

**Published:** 2025-03-26

**Authors:** Lucy L. Mpayo, Haika K. Mariki, Martha Mkony, Karim Manji

**Affiliations:** 1 Department of Pediatric and Child Health, Muhimbili University of Health and Allied Science, P O BOX 65001, Dar Es Salaam, Tanzania; 2 Department of Pediatric and Child Health, Muhimbili National Hospital, P O Box 65000, Dar Es Salaam, Tanzania

**Keywords:** Femoral–facial syndrome, Prenatal folate, Antenatal scanning

## Abstract

**Background:**

Femoral–facial syndrome is a very rare condition that occurs sporadically. Few cases have been linked to maternal diabetes and genetic origin. We report a case of a preterm infant.

**Case report:**

A 1-day-old Black Bantu African female baby was admitted to the neonatal unit owing to multiple congenital anomalies. The mother did not seek any medical attention until her early third trimester when she had an abruptio placenta. The mother has never received antenatal folic acid supplementation.

The baby had microcephaly, low-set ears, right cleft palate and lip, micrognathia, contracture, and shorter limbs. A skeletal X-ray showed femoral hypoplasia.

We report this rare case and highlight the challenges in diagnosis and treatment.

**Conclusion:**

This is an unfortunate occurrence. Early antenatal folic acid supplementation may have averted this. An early antenatal anomaly scan and investigation after abruptio placenta would have shown the futility of the case, and intervention could have been done sooner rather than later.

## Background

Femoral–facial syndrome (FFS, OMIM 134780) is an extremely rare syndrome characterized by multiple congenital anomalies involving the femur, cranial, mid-face, and extremities. Its prevalence has been reported to be less than 1/1,000,000 [1, 2]. It occurs sporadically, however, etiological association with maternal diabetes has been reported. Genetic transmission of this condition is related to the locations of genes on autosomes (dominant) in some families, but a specific gene mutation has not been identified [3, 4]. However, Spielmann *et al*. report a case of FFS associated with a chromosome 2q37 rearrangement [5].

The main features of this syndrome are bilateral femoral aplasia/hypoplasia and cranial–facial dysmorphism, however, a wide range of congenital malformations were described in 1975 [6]. Cranio-facial features comprise micrognathia, low-set ears, broad nose, long philtrum, flat philtrum, cleft palate, thin upper lip, upward slanting eyelids, and craniosynostosis [6, 7]. Other systemic anomalies may also be associated with FFS. Congenital heart diseases, including septal defects and patent ductus arteriosus, are the commonest in FFS [8]. Chest deformities, agenesis, and polycystic kidney may be associated with FFS. Other skeletal manifestation of FFS include scoliosis, long bone synostosis, talipes equinovarus, macrodactyly, and polydactyly [8, 9].

We report herein a case of FFS in a preterm infant.

## Case presentation

A 1-day-old, Black Bantu African preterm female baby weighing 1000 g was born to a Gravida 3, Living 2 mother via spontaneous vaginal delivery. Soon after delivery, she was referred to our neonatal intensive care unit owing to multiple congenital anomalies and moderate respiratory distress. The mother is 32 years and is in a non-consanguineous marriage. The mother did not seek any medical attention until her early third trimester of pregnancy, due to abruptio placenta. The first antenatal ultrasound revealed severe oligohydramnios. She was not diabetic (random blood glucose was 5.1 mmol/L) and did not receive folic acid supplementation during pregnancy.

**Fig. 1:**
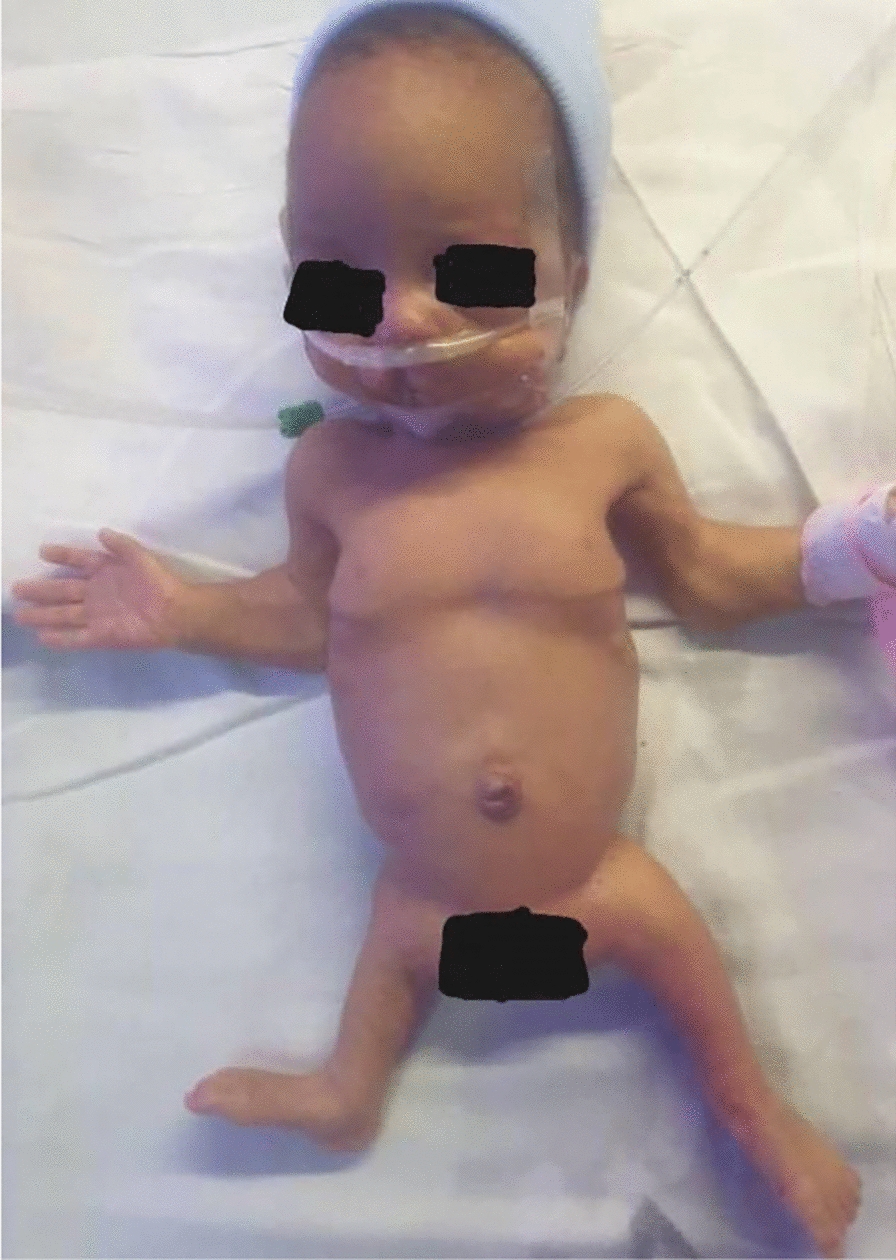
A preterm infant with multiple congenital anomalies including: a small head, unilateral right cleft palate and lip, abnormal chest cavity, elbow contracture, and shorter limbs

At admission, the baby was alert with mild features of respiratory distress syndrome. She had multiple dysmorphic features that included microcephaly (head circumference < 10%), prominent occiput, low-set ears, right cleft palate and lip, micrognathia, elbow contracture bilaterally, and shorter lower limbs (Fig. 1).

The birth weight was 1000 g, the length of the baby was 40 cm, and the occipitofrontal circumference was 30 cm.

## Investigations

**Fig. 2:**
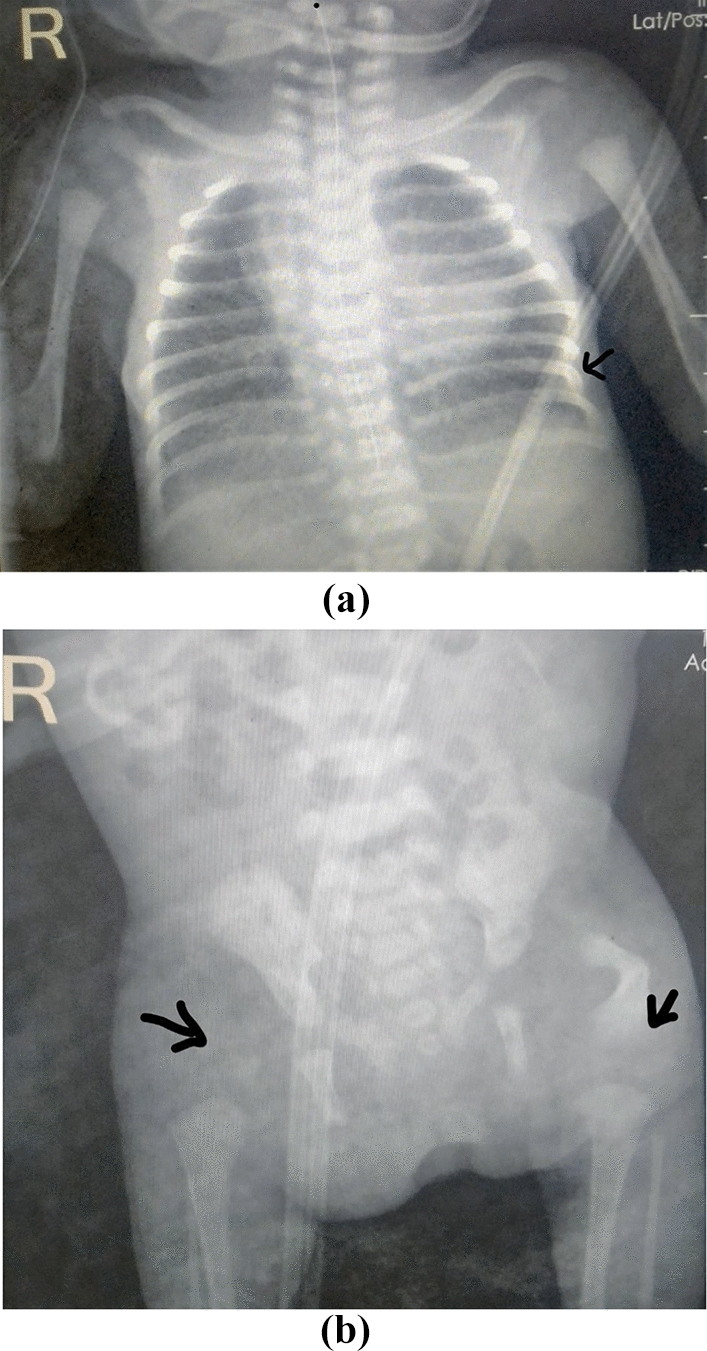
**a** Anterior–posterior view chest X-ray showing rib deformities (arrow). **b** Skeletal X-ray showing right femoral aplasia and left femoral hypoplasia (arrows)

Skeletal X-ray showed rib flattening, right femur aplasia, and left femur hypoplasia (Fig. 2a and b). Laboratory workout included: complete blood counts, c-reactive protein, creatinine, blood urea, and electrolytes, which were all within the normal range. Cranial ultrasound was normal, but echocardiography revealed a small patent ductus arteriosus.

## Differential diagnosis

The patient had bilateral femoral aplasia and unusual facies. These clinical entities led to the diagnosis of femoral–facial syndrome.

The similarity in etiopathogenesis makes caudal regression syndrome the common differential diagnosis. Both can be caused by poorly controlled maternal hyperglycemia. However, facial defects are absent in caudal regression syndrome, the key diagnostic feature.

Proximal focal femoral hypoplasia and craniofacial anomalies are usually absent.

Disruption conditions that can cause cleft palate include severe amniotic band syndrome.

## Treatment

The infant was kept on respiratory support and empirical antibiotics. An orthopedic surgeon, an ear, nose, and throat surgeon, and a physiotherapist were involved. Surgical repair of the cleft palate is planned at age of 6 months. Functional rehabilitation is planned before the patient starts ambulation. Limb lengthening surgery is currently not done in our setting.

## Outcome and follow-up

At the age of 1 month, the patient died from complications of severe late-onset neonatal sepsis.

## Discussion

The clinical entity of FFS has been intensively described in the literature in approximately 70 cases reported to date [1–10]. The criteria for clinical diagnosis of FFS have evolved from femoral hypoplasia plus one or more major craniofacial features, to femoral hypoplasia plus two or more facial anomalies [10].

The etiopathogenesis of FFS is still unknown and most cases are found to occur sporadically. Maternal hyperglycemia is considered to be teratogenic exposure, associated with 38% of cases of FFS [3, 4, 7, 10, 11]. Other possible exposures, including antenatal drugs, maternal infections, and oligohydramnios, have been reported [4]. In our case, the mother was not found to have had hyperglycemia during the third trimester until the time of delivery. To the best of her knowledge, there is no family history of congenital anomalies, however, she booked antenatal care after 28 weeks gestation.

The pathogenesis of caudal dysgenesis in an infant of a diabetic mother is due to an insufficiency of mesoderm [12]. Caudal regression syndrome and FFS have some similarities in etiopathogenesis [11]. The development of the femur during embryogenesis arises from the lateral plate mesoderm, which proliferates and differentiates into chondrocytes. The mother was not tested earlier during pregnancy, but she was euglycemic when she booked for antenatal care at 28 weeks.

Folic acid is very important in folate metabolism during embryogenesis, particularly in cellular proliferation and differentiation [13]. Embryonic tissues are very sensitive to folate deficiency: later contributing to neural tube defects and non-syndromic cleft palate [13, 14]. There is only one case reported of an FFS infant born to a mother who did not consume folic acid during pregnancy, and she was also found to be hyperglycemic during delivery [15]. We, therefore, assume that this may also be the case with our infant.

Depending on various degrees of severity, patients with FFS can reach adulthood, and most are ambulatory with normal intelligence [6, 10]. The problems they encounter include feeding difficulties and speech development [1, 6].

We report an extremely rare case, but also emphasize the importance of antenatal folic acid supplementation and early antenatal care. Large epidemiological studies are required to establish an etiological association between antenatal folic acid supplementation and FFS.

## Conclusion

- Femoral–facial syndrome is an extremely rare condition with multiple congenital anomalies which interfere with quality of life, and termination of pregnancy may not be the only option
- Further epidemiological evidence is required to study the etiological association of antenatal folic acid supplementation and FFS.
- The performance of fetal scans, especially after antepartum hemorrhage, is important to identify potentially life-threatening conditions which precipitate the same.

## Data Availability

Not applicable.
